# Two False Negative Test Results in a Symptomatic Patient with a Confirmed Case of Severe Acute Respiratory Syndrome Coronavirus-2 (SARS-CoV-2) and Suspected Stevens-Johnson Syndrome/Toxic Epidermal Necrolysis (SJS/TEN)

**DOI:** 10.7759/cureus.8198

**Published:** 2020-05-19

**Authors:** Tomer Lagziel, Luis Quiroga, Margarita Ramos, Charles S Hultman, Mohammed Asif

**Affiliations:** 1 Plastic Surgery, Johns Hopkins University School of Medicine, Baltimore, USA; 2 Medicine, Tel-Aviv University, Sackler School of Medicine, Tel-Aviv, ISR

**Keywords:** covid-19, sjs/ten, burn surgery, rt-pcr, patient under investigation, false-negative

## Abstract

The recent outbreak of COVID-19 has put significant strain on the current health system and has exposed dangers previously overlooked. The pathogen known as severe acute respiratory syndrome coronavirus 2 (SARS-COV-2), is notable for attacking the pulmonary system causing acute respiratory distress, but it can also severely affect other systems in at-risk individuals including cardiovascular compromise, gastrointestinal distress, acute kidney injury, coagulopathies, cutaneous manifestations, and ultimately death from multi-organ failure. Unfortunately, the reliability of negative test results is questionable and the high infectious burden of the virus calls for extended safety precautions, especially in symptomatic patients. We present a confirmed COVID-19 case that was transferred to our burn center for concern of Steven Johnson syndrome/toxic epidermal necrolysis (SJS/TEN) overlap syndrome after having two negative confirmatory COVID-19 tests at an outside hospital.

A 58-year-old female with a history of morbid obesity, HTN, gout, CML managed with imatinib, and chronic kidney disease presented as a transfer from a community hospital to our burn center. The patient was admitted to her community hospital with febrile, acute respiratory distress. Imaging and clinical presentation was consistent with COVID-19 and lab tests for the pathogen were ordered. During observation, while waiting for results, she was placed under patient under investigation (PUI) protocol. Once negative results were obtained, the PUI protocol was abandoned despite ongoing symptoms. Subsequently, dermatological symptoms developed and transfer to our burn center was initiated. After a second negative test result, the symptomatic patient was transferred to our burn center for expert wound management. Given the lack of resolve of respiratory symptoms and concern for the burn patient population, the patient was placed in PUI protocol and an internal COVID-19 was ordered. The patient’s initial exam under standard COVID-19 airborne precautions revealed 5% total body surface area of loss of epidermis affecting bilateral thighs, bilateral arms, and face. A dermatopathological biopsy suggested a bullous drug reaction with an erythema multiform-like reaction pattern versus SJS/TEN. Moreover, the internal COVID-19 test returned positive.

The delayed positive test results and complicated hospital course with our patient required us to scale back and notify every patient and staff member whom they came in contact with, across multiple institutions. We suggest that whenever a suspected COVID-19 patient is transferred to a specialized center, they should be isolated and re-checked before joining the new patient population for treatment of the unique condition.

## Introduction

The world is in the midst of a pandemic led by the recent outbreak of a novel coronavirus. The new pathogen is known as the severe acute respiratory syndrome coronavirus 2 (SARS-COV-2) and by its official World Health Organization (WHO) disease name, COVID-19 [1]. Although, this virus is notable for attacking the pulmonary system, causing acute respiratory distress syndrome (ARDS), it can also severely affect other systems in at-risk individuals including cardiovascular compromise, gastrointestinal distress, acute kidney injury, coagulopathies, cutaneous manifestations and ultimately death from multi-organ failure [2-3]. At present, April 2020, the United States has surpassed every country in the number of active cases, with over 900,000 cases and approximately 51,000 confirmed deaths since it was announced as a pandemic in late January [4]. Currently, the gold-standard for quick confirmation of COVID-19 is a real-time reverse-transcriptase polymerase chain reaction (rRT-PCR) [5]. Unfortunately, this test is not 100% accurate and early reports from China suggest a false-negative result rate of up to 20% [6-8]. The high infectious burden of COVID-19 brings into question the reliability of negative test results when managing high-risk patient populations. We present a COVID-19 case that was transferred to our burn center for concern of Steven Johnson syndrome/toxic epidermal necrolysis (SJS/TEN) overlap syndrome after having two negative confirmatory COVID-19 tests at an outside hospital.

## Case presentation

A 58-year-old female with a history of morbid obesity, hypertension (HTN), gout, chronic myeloid leukemia (CML) managed with imatinib for four years, and chronic kidney disease (CKD), presented as a transfer from a community hospital with suspected SJS/TEN overlap syndrome. The patient was prescribed levofloxacin and oseltamivir by her primary care physician for complaints of one week of coughing, fevers, and fatigue, despite having negative influenza testing. After one week, she presented to her local emergency room with worsening respiratory symptoms, a fever of 100.8^o^F, and 90% O_2_ saturation on room air. A chest radiograph (CXR) and chest computed tomography (CT) scan demonstrated multifocal pneumonia. The patient was immediately placed under patient under investigation (PUI) protocol, broad-spectrum antibiotics including vancomycin, and piperacillin and tazobactam were started after obtaining blood cultures and a COVID-19 test. The hospital course was complicated by acute kidney injury (AKI) with creatinine levels of 2.8 mg/dL that peaked 7.9 mg/dL prior to dialysis intervention. Six days later, the COVID-19 test results were negative and the PUI protocol was abandoned despite ongoing respiratory distress. Her pulmonary symptoms continued to be managed with antibiotic and supportive therapy. The following day, during routine physical examination in the general ward, the patient was noted to have a disseminated erythematous and papular skin rash. Prophylactic hydrocortisone therapy was initiated for a suspected allergic reaction, especially concerning given her pulmonary symptoms, and antibiotics were held due to possible drug reaction. 

During the next 48 hours, the rash developed into vesicles and bullae with desquamation, forming widespread, large, open wounds. SJS/TEN was suspected and her treatment team requested to transfer her to our tertiary care burn center for expert wound care and management. Given her persistent respiratory distress, a second COVID-19 test was rushed and administered prior to transfer which also returned negative and the patient transfer was processed after 24 hours.

**Figure 1:**
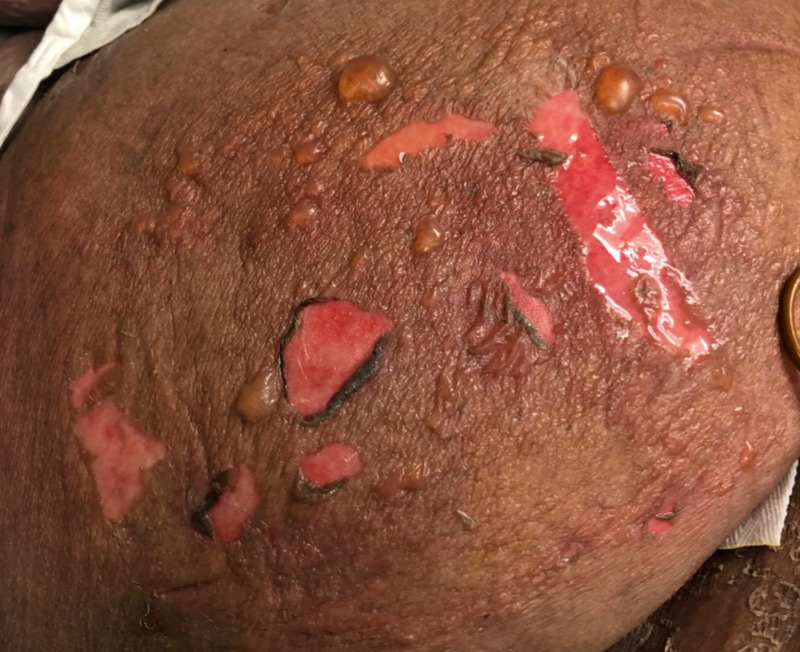
Bullae and positive Nikolsky’s sign on the lateral aspect of the patient's right thigh one day after admission

The patient arrived at our hospital in severe respiratory distress concomitant with blistering dermatological lesions. Given that treating the patient in the burn unit requires exposure to an immunocompromised patient population, a decision was made to place the patient in the surgical intensive care unit (SICU) for PUI protocol in a negative pressure room. The patient’s initial exam under standard COVID-19 airborne precautions revealed 5% total body surface area of epidermal loss affecting bilateral thighs, bilateral arms, and face (Figure 1). 

**Figure 2:**
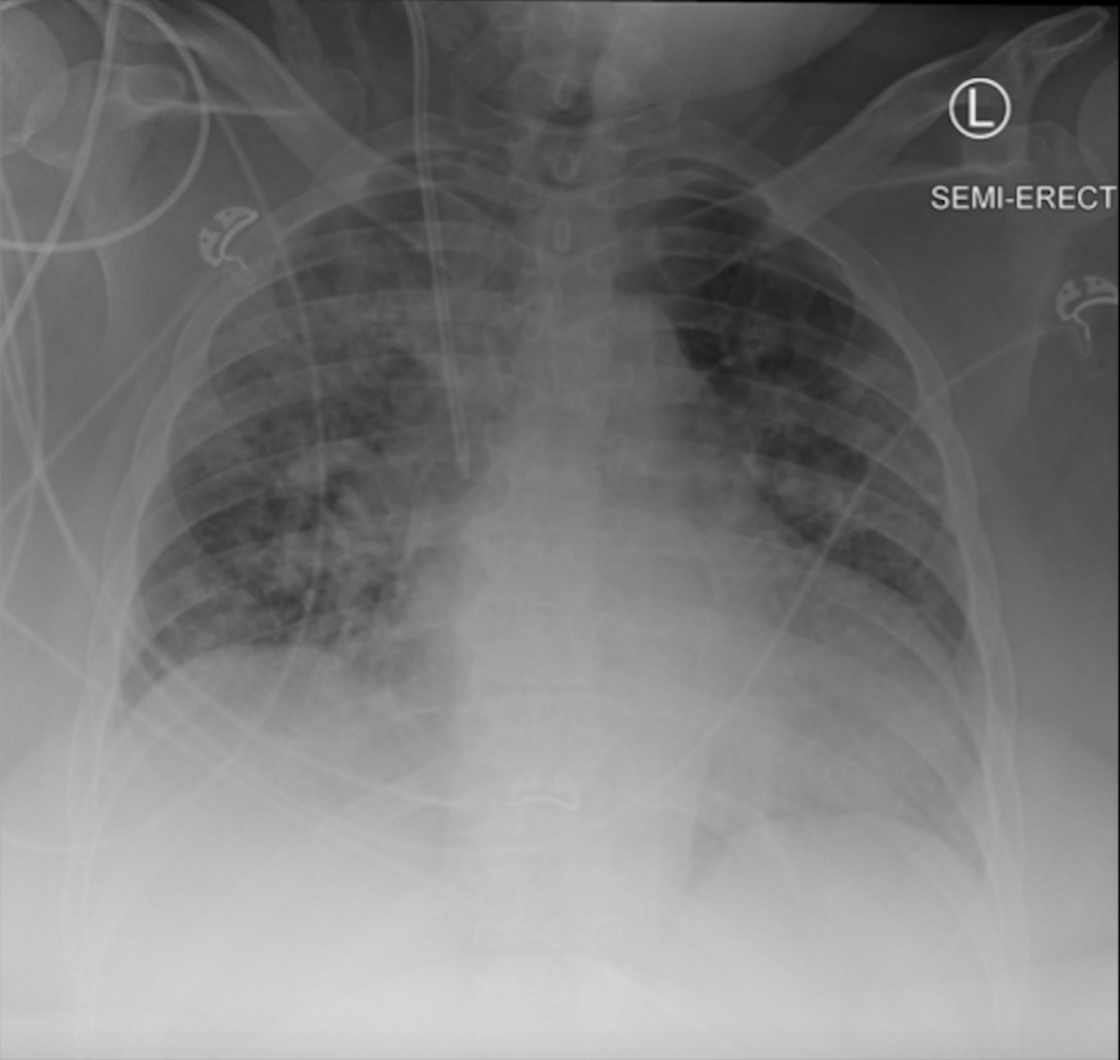
Chest radiograph obtained at our institution on the day of the patient's transfer from the outside hospital

The combined risk of the uncertain validity of external COVID-19 tests, the severe symptomatic nature of the patient, and the vulnerable burn unit patient population prompted the decision to order a third, internal, COVID-19 test, and to obtain an additional CXR (Figure 2).

A dermatopathological biopsy suggested a bullous drug reaction with an erythema multiform-like reaction pattern versus SJS/TEN. The biopsied sections demonstrated detached epidermis with a "basket-weave" stratum corneum that is separated at the dermal-epidermal junction. There were spongiosis and subtle basilar vacuolar changes with rare dyskeratotic cells. The dermis demonstrated superficial edema and a mildly dense, superficial, perivascular, and interstitial infiltrate composed of lymphocytes and histiocytes with occasional eosinophils and melanophages. The final dermatological clinical picture with no oral mucosal involvement was most consistent with resolving bullous interface dermatitis, not SJS/TEN. The skin lesions were treated with local wound care using silver antimicrobial foam dressing, changed twice a week with the patient placed on a taper of oral prednisone. The COVID-19 test results, however, returned positive after 48 hours. Subsequently, the patient’s hemodynamic status improved and she was transferred to a designated regular COVID-19 unit floor and days later discharged to a skilled nursing facility for further wound care and management. The patient was able to finish the prednisone treatment as an outpatient successfully and recover from COVID-19.

## Discussion

The 2019 novel coronavirus is still in the early stages of medical investigation and as pulmonary management advances, we begin to examine the multi-system manifestations of this deadly contagion. The previous global spreads of severe acute respiratory syndrome (SARS) and the Middle East respiratory syndrome (MERS) have demonstrated the respiratory and epidemiologic manifestations of COVID-19, but also has demonstrated that this new viral disease has unique characteristics [9]. Little peer-reviewed literature is available on the dermatologic symptoms of SARS-CoV-2. However, a review of 88 COVID-19 positive patients in Lombardo, Italy, reported 18 patients with cutaneous manifestations, none of whom were suspected for SJS/TENS [10]. The broad range of dermatologic symptoms suggests an unclear pathologic mechanism. A hematologic review of immunosuppressive medications was performed in Italy to examine their therapeutic role in the setting of COVID-19 and their findings suggest a possible advantage of imatinib, for CML, in fighting COVID-19 [11]. Skin adverse effects are common in imatinib users (especially women), but generally appear within one month of beginning therapy [12-13]. Given these findings, the relationship between skin findings in our patient and imatinib is unclear but the drug's presence could have even played a role in the patient's resolution. It has been recently reported that genetic predisposition, given specific polymorphisms, could play a significant role in autoimmune/inflammatory manifestations of COVID-19 [14]. While no petechiae or purpuric manifestations were noted in our patient, recent correspondence from the New England Journal of Medicine reported a patient with immune thrombocytopenic purpura (ITP) as a result of COVID-19 [15]. The mechanism for this manifestation is unclear but we suggest the cutaneous symptoms in our patients could have resulted from paradoxical cutaneous micro-emboli and hypercoagulation. Treatment of skin drug reactions has the additional challenge of the unknown effects of systemic steroids, which has been a mainstay of treatment for these conditions in COVID-19 positive patients [16]. In the case presented, the patient received oral steroids which were tapered over a week and had concurrent resolution of COVID-19 disease.

## Conclusions

COVID-19 has rapidly become a significant burden on the global health system due to its unknown characteristics and disease severity in at-risk individuals. Because of the extreme dangers associated with this virus along with many unknown variables, we advise that every suspected hospitalized case should be treated as a potential COVID-19 case, and that the interpretation of any test should follow, as always, the clinical judgment. Our report exemplifies this, because most of the patients in the burn unit are in an immunocompromised state whether due to medications or the severe burn wounds. In addition, the burn unit lacks negative pressure rooms suited for COVID-19 patients. If the patient had not been transferred to the SICU, but directly to the burn center, they would have placed the entire floor and staff at risk of infection. Due to the extreme infective burden of COVID-19, protecting healthcare staff and at-risk patients should be a priority. The delayed positive test results and complicated hospital course with our patient required us to scale back and notify every patient and staff member that they came in contact with across multiple institutions. We suggest that whenever a suspected COVID-19 patient is transferred to a specialized center, they should be isolated and re-checked before joining the new patient population for treatment of the unique condition. In addition, as long as symptoms remain, the suspected patient should be maintained in a PUI protocol because the high false-negative rate of rRT-PCR testing can put patients and staff at risk.
